# Potential role of FoxO1 and mTORC1 in the pathogenesis of Western diet-induced acne

**DOI:** 10.1111/exd.12142

**Published:** 2013-04-25

**Authors:** Bodo C Melnik, Christos C Zouboulis

**Affiliations:** 1 Department of Dermatology, Environmental Medicine and Health Theory, University of OsnabrückOsnabrück, Germany; 2 Departments of Dermatology, Venereology, Allergology and Immunology, Dessau Medical CenterDessau, Germany

**Keywords:** acne, FoxO1, mTORC1, nutrient signalling, Western diet

## Abstract

Acne in adolescents of developed countries is an epidemic skin disease and has currently been linked to the Western diet (WD). It is the intention of this viewpoint to discuss the possible impact of WD-mediated nutrient signalling in the pathogenesis of acne. High glycaemic load and dairy protein consumption both increase insulin/insulin-like growth factor-1 (IGF-1) signalling (IIS) that is superimposed on elevated IGF-1 signalling of puberty. The cell's nutritional status is primarily sensed by the forkhead box transcription factor O1 (FoxO1) and the serine/threonine kinase mammalian target of rapamycin complex 1 (mTORC1). Increased IIS extrudes FoxO1 into the cytoplasm, whereas nuclear FoxO1 suppresses hepatic IGF-1 synthesis and thus impairs somatic growth. FoxO1 attenuates androgen signalling, interacts with regulatory proteins important for sebaceous lipogenesis, regulates the activity of innate and adaptive immunity, antagonizes oxidative stress and most importantly functions as a rheostat of mTORC1, the master regulator of cell growth, proliferation and metabolic homoeostasis. Thus, FoxO1 links nutrient availability to mTORC1-driven processes: increased protein and lipid synthesis, cell proliferation, cell differentiation including hyperproliferation of acroinfundibular keratinocytes, sebaceous gland hyperplasia, increased sebaceous lipogenesis, insulin resistance and increased body mass index. Enhanced androgen, TNF-α and IGF-1 signalling due to genetic polymorphisms promoting the risk of acne all converge in mTORC1 activation, which is further enhanced by nutrient signalling of WD. Deeper insights into the molecular interplay of FoxO1/mTORC1-mediated nutrient signalling are thus of critical importance to understand the impact of WD on the promotion of epidemic acne and more serious mTORC1-driven diseases of civilization.

## Introduction

Acne is a disease of Western civilization with prevalence rates in adolescence of over 85% 1–3. Western diet (WD), characterized by high glycaemic load and high dairy protein consumption, has been suggested to be a fundamental nutritional factor promoting the acne epidemic 4,5. Proper functioning of the pathways that are involved in sensing of nutrients is central to metabolic homoeostasis 6. Notably, acne is absent in populations consuming less insulinotropic Palaeolithic diets 1,7, which exclude grains, milk and dairy products and exhibit much lower insulin/insulin-like growth factor (IGF-1) signalling (IIS) 4,7.

It is the purpose of this viewpoint to elucidate the molecular pathology of nutrient signalling of WD in the pathogenesis of acne. WD-derived metabolic signals are sensed by the forkhead box class O1 transcription factor (FoxO1) and the nutrient-sensitive kinase mammalian target of rapamycin complex 1 (mTORC1). mTORC1 is regarded as the conductor of the ‘cellular signalling symphony’ that integrates signals of cellular energy, growth factors and amino acids 8,9. Metabolic regulations mediated by FoxO1 and mTORC1 depend on upstream activation of the IIS cascade, required for adaptive nutrient homoeostasis and endocrine growth regulation 10.

## Western diet upregulates insulin/IGF-1 signalling

The major endocrine changes of puberty primarily depend on hepatic secretion of IGF-1, the principal mediator of somatic growth promoting sebaceous gland (SG) cell proliferation and lipogenesis 11–17. WD significantly increases insulin and IGF-1 serum levels and thus exaggerates already upregulated IIS of puberty 4,18.

**Figure 1:**
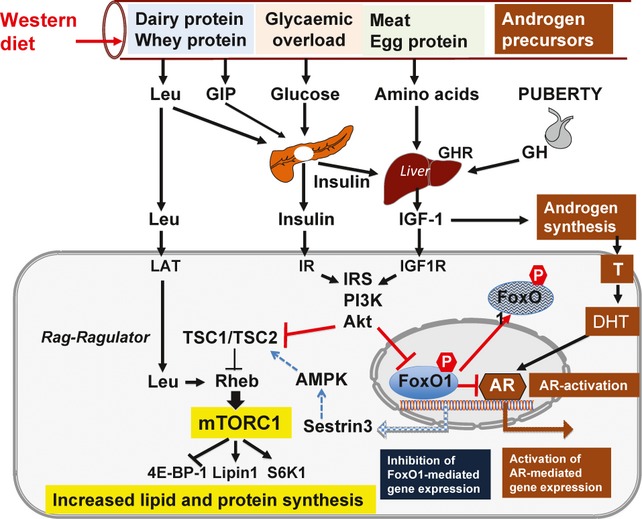
Increased insulin/IGF-1 signalling (IIS) of Western diet (WD) results in Akt-mediated FoxO1 inhibition by nuclear extrusion. Akt-mediated phosphorylation of TSC2 attenuates the inhibitory effect of TSC1/TSC2 on Rheb, thus promotes mTORC1 activation. In contrast, nuclear activation of FoxO1 stimulates the expression of sestrin3, which via AMPK activation inhibits mTORC1. Increased IIS of WD is superimposed on enhanced IIS of puberty, thereby promotes the development of acne. FoxO1 inhibits GHR expression, hepatic IGF-1 synthesis and androgen receptor (AR) transactivation. GIP, glucose-dependent insulinotropic polypeptide; GH, growth hormone; GHR, GH receptor; Leu, leucine; LAT, L-type amino acid transporter; IR, insulin receptor; IRS, insulin receptor substrate; PI3K, phosphoinositol-3 kinase; Akt, Akt kinase (protein kinase B); FoxO, forkhead box transcription factor class O; TSC, tuberous sclerosis complex; Rheb, ras-homolog enriched in brain; mTORC1, mammalian target of rapamycin complex 1; AMPK, AMP kinase; T, testosterone; DHT, dihydrotestosterone.

Placebo-controlled studies have demonstrated that high glycaemic load diets aggravate acne, result in postprandial hyperinsulinaemia and increase serum levels of free IGF-1 19–27. Epidemiological as well as clinical evidence confirmed that milk and other insulinotropic dairy products induce or aggravate acne 23,24,28–34. Whey protein abuse by athletes and bodybuilders has recently been reported to induce acne flares 33,34. Milk is not a ‘simple food’ but has been identified as an endocrine growth–promoting signalling system of mammals, which activates mTORC1 signalling but inhibits FoxO1-dependent gene regulation 35 (Fig. 1).

## FoxO1: a nutrient-sensing transcription factor

**Figure 2:**
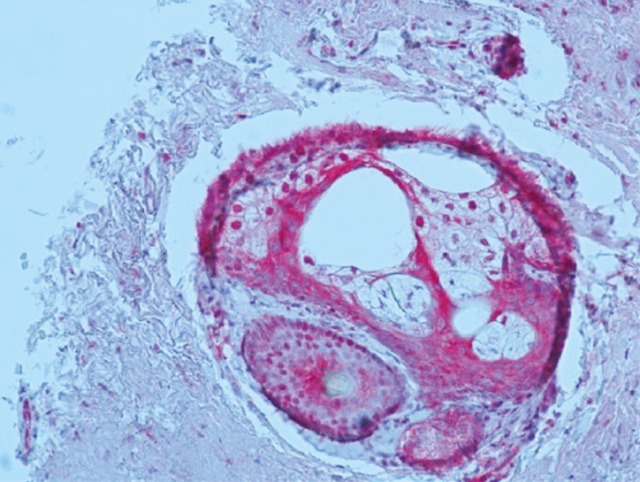
Immunohistochemical detection of FoxO1 in human sebaceous glands (kindly provided by Dr. A. I. Liakou, Dessau Medical Center, Germany)

FoxO1 is an important transcription factor that modulates the expression of genes involved in cell cycle control, DNA damage repair, apoptosis, oxidative stress management, cell differentiation, glucose and lipid metabolism, inflammation, and innate and adaptive immune functions 36–42. FoxO1 is expressed in all mammalian tissues including human SGs (Fig. 2) and plays an important role in the regulation of metabolism 43. Mouse hepatic chromatin exhibited 401 FoxO1-binding locations, regulating metabolic processing of carboxylic acids, fatty acids, steroids and retinoids 44. FoxO1 has been proposed to function as a key regulator in the pathogenesis of acne as FoxO1 senses external nutrient and internal growth factor signals and relays these to FoxO1-dependent gene regulation 45.

Central to the regulation of FoxOs is their shuttling either into the nucleus or into the cytosol. FoxO1 is inhibited by its export into the cytoplasm, which requires specific phosphorylation of FoxO1 in the nucleus by activated Akt kinase 36,37,40. The phosphoinositol-3 kinase (PI3K)/Akt cascade is stimulated by growth factors like insulin and IGF-1 and is negatively regulated by the phosphatase PTEN 46. Thus, increased IIS of WD is superimposed on enhanced IIS of puberty, the two converging in inhibition of FoxO1-dependent gene regulation 4 (Fig. 1).

## FoxO1 inhibits hepatic IGF-1 secretion

Nuclear FoxO1 is highly upregulated during fasting. However, in the postprandial state and nutrient overload, enhanced IIS inhibits FoxO1 43,47,48. It is understandable that FoxO1 closely interacts with regulators of somatic growth, which is suppressed in the absence of nutrients. A major regulatory node of somatic growth and hepatic IGF-1 secretion is the growth hormone receptor (GHR).

Untreated individuals with Laron syndrome, a primary growth hormone (GH) resistance disorder due to a genetic defect of GHR, exhibit diminished congenital IGF-1 serum levels, are of short stature and never develop acne 49,50. IGF-1-deficient serum of Laron individuals increased nuclear FoxO levels and inhibited mTORC1 activity 50. The DKO mouse is an animal model mimicking Laron syndrome with impaired IIS. DKO mice have a double knockout of insulin receptor substrate-1 (IRS)-1 and IRS-2 in the liver, are shorter and exhibit 20% less body mass than control mice 10. Reduced hepatic Akt signalling with increased nuclear FoxO1 levels in DKO mice resulted in decreased expression of GHR, IGF-1 and sterol regulatory element binding protein (SREBP)-1c and increased expression of IGF-binding protein-1 (IGFBP-1) 10.

It has recently been confirmed in mice that GHR and IGFBP-1 are FoxO1 target genes 44. FoxO1-mediated inhibition of GHR expression in the liver attenuates GH-mediated synthesis and secretion of hepatic IGF-1, the main source of IGF-1 in the systemic circulation 10. Furthermore, FoxO1 induces hepatic expression of circulating IGFBP-1, thereby reducing the bioavailability of free IGF-1 10,51,52. Reduced levels of IGF-1 attenuate both general growth and SG growth and lipid synthesis 13–17.

Cell growth is controlled by cell cycle inhibitors. Notably, FoxO1 activates the expression of the cell cycle inhibitors p21 and p27 (see Data S1) 53–58. Moreover, FoxO1 activates the expression of the eukaryotic initiation factor 4E-binding protein-1 (4E-BP-1), which is a major substrate of mTORC1 and functions as a potent translational inhibitor and growth suppressor (Table 1) 59,60.

**Table 1:** Important FoxO1-regulated target genes in the pathogenesis of acne

Growth hormone receptor (GHR)	Suppression of GHR expression with downregulation of hepatic IGF-1 synthesis
IGF-binding protein-1 (IGFBP-1)	Upregulation of IGFBP-1 expression, reduction in circulating free IGF-1
Eukaryotic initiation factor 4 binding protein-1 (4E-BP-1)	Activation of 4E-BP-1 expression inhibiting mRNA translation
p21	Activation of p21 expression, cell cycle inhibition, growth inhibition
p27	Activation of p27, cell cycle inhibition, growth inhibition
Sestrin3	Activation of sestrin3 expression, activation of AMPK-mediated phosphorylation of TSC2 activating the inhibitory function of TSC1/TSC2, thus suppressing mTORC1
Haeme oxygenase-1 (OH-1)	Activation of OH-1 expression, inhibition of mitochondrial function and reactive oxygen species formation, inhibition of NFκB, inhibition of inflammation

## FoxO1 inhibits lipogenesis

FoxO1 not only suppresses protein synthesis and cell growth, but also lipid metabolism. FoxO1 regulates the key transcription factor of lipid synthesis SREBP-1c (Table 1) 10,61. IGF-1 induced SREBP-1 expression and enhanced lipogenesis in SEB-1 sebocytes via activation of the PI3K/Akt pathway 17, whereas FoxO1 antagonized the expression of SREBP-1c 10,61. Thus, reduced expression of SREBP-1 should be expected from a low glycaemic load diet associated with attenuated IIS 4. In fact, Kwon et al. 25 demonstrated that a 10-week low glycaemic load diet reduced SREBP-1 expression in the skin of acne patients, reduced the size of SGs, mitigated cutaneous inflammation and improved acne. Furthermore, FoxO1 suppresses the activity of peroxisome proliferator–activated receptor-γ (PPARγ) and LXRα 61–64 that both costimulate SG lipogenesis (Table 2) 65–70.

**Table 2:** FoxO1 interaction with regulatory proteins and transcription factors

Androgen receptor (AR)	Suppression of AR transactivation
PPARγ	Suppression of PPARγ and PPARγ-mediated lipogenesis
LXRα	Suppression of RXR/LXRα-mediated activation of SREBP-1
TSC2	Akt-phosphorylated cytoplasmic FoxO1 dissociates and thereby inhibits the TSC1/TSC2 heterodimer
β-Catenin	Augmentation of nuclear FoxO1 signalling
GSK3	Modulation of GSK3-TSC2-mTORC1 signalling
CRM1	Nuclear FoxO1 export

Isotretinoin's sebum-suppressive effect has recently been associated with upregulated FoxO1 expression 71. Reported reductions in IGF-1 serum levels during isotretinoin treatment 72 are thus well explained by FoxO1-mediated inhibition of hepatic GHR expression resulting in diminished hepatic synthesis of IGF-1.

## FoxO1 suppresses androgen signalling

Sebaceous gland growth and acne are androgen dependent 73. The growth of androgen-responsive tissues is coordinated with general somatic growth 74. IGF-1 stimulates gonadal and adrenal androgen synthesis as well as intracutaneous intracrine conversion of testosterone to tenfold more active dihydrotestosterone, the most potent androgen receptor (AR) ligand 11 (Fig. 1). Enhanced hepatic IGF-1 synthesis by WD may thus increase the availability of potent androgens in the skin.

Remarkably, only a few acne patients exhibit hyperandrogenaemia, a fact that points to the predominance of peripheral tissue-dependent androgen/AR sensitivity for the manifestation of acne 73. Intriguingly, FoxO1 functions as an AR cosuppressor 75–77. Nuclear extrusion of FoxO1 by high IIS relieves FoxO1-mediated repression of AR transactivation. Thus, insulinotropic WD may stimulate AR-mediated signalling, which explains enhanced peripheral androgen responsiveness (Fig. 1).

Both AR and IIS synergistically increase SREBP-1-mediated lipogenesis and upregulate lipogenic pathways 78. Whereas FoxO1 stimulates p21 and p27 expression (see Data S1), AR signalling rapidly reduces p27 by increasing its proteasome-mediated degradation 79. These findings exemplify the nutrient-dependent crosstalk between AR and IIS.

## FoxO1 reduces oxidative stress

Overnutrition and anabolic states with enhanced mTORC1 activity are associated with increased oxidative stress, which has been observed in acne vulgaris 80–83. FoxOs upregulate defense mechanisms against reactive oxygen species (ROS). FoxO1 induces the expression of haeme oxygenase 1 and thereby reduces mitochondrial ROS formation 43,84. FoxO1 and FoxO3 mediate the expression of the ROS scavenger sestrin3 (Fig. 1). FoxO3 stimulates the expression of ROS-degrading enzymes manganese superoxide dismutase and catalase 85–87. Hence, FoxOs are key players of redox signalling and link WD to enhanced metabolic oxidative stress in acne vulgaris 81,82,87.

## FoxO1 links nutritional status to innate and adaptive immunity

FoxO family members suppress the highly substrate- and energy-dependent process of T-cell activation 38, whereas FoxO1 deficiency *in vivo* resulted in spontaneous T-cell activation and effector differentiation 39. Increased CD4+ T-cell infiltration and enhanced IL-1 activity have been detected in acne-prone skin areas prior to comedo formation 88. Thus, FoxO1 links nutrient availability and metabolic conditions to T-cell homoeostasis 89,90 (see Data S1).

## FoxOs control antimicrobial peptide synthesis

In *Drosophila* flies, dFOXO controls the expression of several antimicrobial peptides (AMPs) in the skin 91. AMP induction is lost in *dFOXO* null mutants but enhanced when dFOXO is overexpressed. In *Drosophila*, AMP activation can be achieved independently of pathogen-dependent pathways, indicating direct cross-regulation between metabolism and innate immunity 91. As in *Drosophila*, downregulated FoxO inhibited AMP expression in the polyp *Hydra vulgaris* 92.

Downregulated FoxO signalling by WD may thus favour an AMP-deficient follicular microenvironment, which may allow overgrowth of *P. acnes*. WD would not only overstimulate sebum production favouring *P. acnes* growth but may diminish AMP-controlled host responses against *P. acnes,* which may ultimately stimulate inflammatory TLR-mediated innate immune responses against hypercolonized *P. acnes* 93,94. Upregulated TLR-driven innate immune responses against *P. acnes* with overexpression of TNF-α may further enhance SG lipogenesis via activated proinflammatory IKKβ-TSC1-mTORC1 signalling (see Data S1, Fig. S1) 95.

## mTORC1: Convergence point of nutrient signalling in acne

mTORC1 is an evolutionarily conserved nutrient-sensing kinase that regulates growth and metabolism in all eukaryotic cells 8,9,83. mTORC1 signalling serves as a ‘growth checkpoint’ surveying the status of the extra- and intracellular milieu of growth factors and nutrients 83,96. mTORC1 signalling stimulates gene transcription, translation, ribosome biogenesis, protein synthesis, cell growth, cell proliferation and lipid synthesis but suppresses autophagy 8,97–101. In mammalian cells, two functionally different mTOR complexes exist: mTORC1 and mTORC2. mTORC1 contains the partner protein Raptor that interacts with mTORC1 substrates for their phosphorylation. mTORC2 contains the protein Rictor and activates the kinase Akt. mTORC1 controls the G1/S transition and G2/M progression of the cell cycle 98. The mTORC1 signalling network senses and relays diverse inputs of nutrients, growth factors and cellular energy to a central ‘signalling core’ that consists of Akt, TSC1/TSC2, Rheb and mTORC1 itself (Fig. 1) 82,102. Liver kinase B1 and the energy sensor AMP-activated protein kinase (AMPK) are critical regulators of mTORC1 103 (see mTORC1 nutrisome signalling, Data S1).

Western diet overactivates mTORC1 by providing an abundance of dairy- and meat-derived essential amino acids, increased IIS induced by dairy protein consumption and high glycaemic load and suppressed AMPK activity by calorie excess. As protein and lipid biosynthesis, cell growth and proliferation are coordinated by mTORC1, it is obvious that mTORC1 plays a key role in acne pathogenesis, characterized by increased proliferation of acroinfundibular keratinocytes, SG hyperplasia and increased SG lipogenesis 5.

## Acne and mTORC1-driven insulin resistance

Nutrient signalling of WD results in increased activation of downstream substrates of mTORC1, the S6 kinases S6K1 and S6K2. S6K1-mediated phosphorylation of insulin receptor substrate 1 (IRS-1) downregulates IIS and thus induces insulin resistance. Dietary fatty acids directly activate S6K1 independent of mTORC1 104. Insulin resistance is considered to be a physiological feature of increased growth during puberty 105. However, pathologically persistent insulin resistance is associated with the metabolic syndrome as well as acne-associated syndromes 106,107. Thus, increased mTORC1/S6K1 signalling explains the reported associations between WD, acne, increased body mass index (BMI) and insulin resistance 32,108,109.

## mTORC1 regulates lipid synthesis

Increased SG lipid biosynthesis is responsible for seborrhoea and SG hyperplasia. Importantly, the key transcription factor of lipid biosynthesis SREBP-1 depends on mTORC1 activation 110. mTORC1 phosphorylates lipin-1, which controls the access of SREBP-1 to the promoter region of SREBP-1-dependent lipogenic genes in the nucleus 110.

## FoxO1: the rheostat regulating mTORC1

As both mTORC1 and FoxO1 integrate nutrient and growth factor signals, it is conceivable that they interact with each other to coordinate cellular responses to nutrient availability. FoxOs are pivotal inhibitors of mTORC1 and have emerged as important rheostats that modulate the activity of Akt and mTORC1 111. FoxO1, FoxO3 and FoxO4 induce the expression of sestrin3 that activates AMPK, which inhibits mTORC1 (Fig. 1) 85. Activated AMPK phosphorylates FoxO3 and facilitates its nuclear localization 112. Furthermore, Akt-phosphorylated cytoplasmic FoxO1 binds to TSC2 and thereby dissociates the TSC1/TSC2 complex, which activates mTORC1 113. Thus, activated Akt inhibits FoxO1, FoxO3 and FoxO4 through direct phosphorylation and indirectly activates mTORC1, which in turn increases protein and lipid synthesis and induces insulin resistance 113.

mTORC1-activated S6K1 via inhibitory IRS-1-phosphorylation elicits a negative feedback loop to inhibit Akt. In contrast, FoxO1 also induces insulin and IGF-1 receptors and IRS-2, a feedback mechanism, which increases insulin sensitivity 111,114. FoxO1 elevates the expression of Rictor, leading to increased mTORC2 activity that consecutively activates Akt.

Taken together, FoxOs maintain homoeostatic balance between Akt, mTORC1 and mTORC2 (Table 3) 85,111. FoxO1 raises the expression of 4E-BP1, a potent repressor of mRNA translation and suppressor of cell growth 59,60. FoxO1 suppresses the expression of the pseudokinase tribbles 3, which inhibits Akt activity 115,116. FoxO3 elevates the expression of the autophagy-related gene Bnip3 that inhibits mTORC1 117,118. FoxO3 induces the expression of TSC1 and thereby inhibits mTORC1 (Table 1) 119.

**Table 3:** Impact of FoxOs in the regulation of mTORC1 activity

nFoxO1↑	GHR↓, hepatic IGF-1↓, Akt↓	mTORC1↓
nFoxO1↑	IGFBP-1↑, free IGF-1↓, Akt↓	mTORC1↓
nFoxO1↑, nFoxO3↑, nFoxO4↑	Sestrin3↑, AMPK↑, TSC2↑	mTORC1↓
nFoxO1↑	AR↓, mTORC2↓, Akt↓ AR↓, LAT↓, Regulator↓	mTORC1↓
nFoxO1↑	4E-BP-1↑	mTORC1↓
nFoxO1↑	Rictor↑, mTORC2 assembly↑	mTORC1↓
nFoxO1↑	Trb3↑, Akt↓	mTORC1↓
nFoxO3↑	Bnip3↑, Rheb↓	mTORC1↓
nFoxO3↑	FoxO1↑ TSC1↑	mTORC1↓
cFoxO1↑	TSC1/TSC2↓, Rheb↑	mTORC1↑

nFoxO, nuclear FoxO; cFoxO, cytoplasmic FoxO; LAT, L-type amino acid transporter.

In summary, FoxO transcription factors, especially FoxO1, inhibit the activity of mTORC1 at multiple levels of cellular regulation (Table 3).

## Conclusion

Insulinotropic WD impairs FoxO1-mediated gene regulation in acne 45. FoxO1 controls the somatotropic axis, modifies the magnitude of AR signalling, interacts with important nuclear regulators of SG homoeostasis, metabolism and lipogenesis and most importantly coordinates the activity of mTORC1.

Acne vulgaris with exacerbated pilosebaceous mTORC1 signalling belongs to the family of mTORC1-driven diseases of civilization 5. Dermatologists counselling acne patients, especially the young, should not only focus on the treatment of skin pathology but should advise on means to correct inappropriate systemic mTORC1 signalling that is aggravated by WD. This is essential to prevent more serious mTORC1-driven diseases of civilization like obesity, diabetes and cancer 5,120.

Nutritional therapy of acne should (i) normalize total calorie intake, (ii) lower glycaemic load and (iii) restrict total dairy protein consumption, especially whey protein abuse 5,19,25,32–34. The ideal nutritional therapy of acne should favour (i) a *Palaeolithic-type diet* containing less insulinotropic grains and minimal or no dairy products to avoid increased IIS and androgen precursors present in dairy products, (ii) higher consumption of vegetables, fruits and green tea containing natural plant-derived mTORC1 inhibitors (epigallocatechin gallate, resveratrol and other natural polyphenols) 121 and (iii) increased consumption of fish (lower insulinaemic index than dairy protein; source of anti-inflammatory ω-3 fatty acids) and adequate intake of vitamin D (see Data S1, Fig. S1).

Deeper insights into the regulation of nutrient signalling may help dermatologists to understand the central role of WD in the pathogenesis and treatment of acne and may shed a new light on the precious experience of Hippocrates of Kos who stated about 2400 years ago: ‘*Your diet should be your medicine, and your medicine should be your diet’*.

## Supporting Information

**Data S1.** Further FoxO1/mTORC1 interactions.

**Figure S1.** Interaction of acne risk-enhancing gene polymorphisms on FoxO1-/mTORC1 mediated nutrient signalling of Western diet.
