# Supplementary material for: Potential role of FoxO1 and mTORC1 in the pathogenesis of Western diet-induced acne
Source: Exp Dermatol. 2013 Apr 25;22(5):311–5. doi: 10.1111/exd.12142 (PMC3746128; doi:10.1111/exd.12142)
Supplement: Supplementary file 1 [file exd0022-0311-SD1.doc]

**Data S1: Further FoxO1/mTORC1 interactions**

**Major regulatory pathways of nutrisome signalling**

The activation of mTORC1 depends on two parallel mechanisms: 1) upstream activation of the small GTPase Rheb (Ras homolog enriched in brain) by growth factor signals and high cellular energy levels, and 2) amino acid (AA)-dependent translocation of inactive mTORC1 to active Rheb localized in lysosome compartments (122-124). The activity of Rheb is tightly regulated by the tuberous sclerosis proteins TSC1 (hamartin) and TSC2 (tuberin), which form a functional heterodimeric complex. TSC1 stabilizes TSC2, which possesses a GTPase-activating protein that hydrolyses GTP to GDP. The TSC1/TSC2 complex provides this function to Rheb leading to inactivation of Rheb. IIS, via activated Akt as well as other growth-related kinases such as ERK and RSK, phosphorylate TSC2 and thereby suppress the inhibitory function of the TSC1/TSC2 complex. IKKβ, the crucial kinase of proinflammatory NFκB activation, phosphorylates and thereby inhibits TSC1 (125, 126). The inhibition of either TSC1 or TSC2 leads to activation of Rheb and ultimately of mTORC1 (127-129).

AMPK plays a key role in energy-dependent regulation of mTORC1. AMPK is activated during energy-deficient conditions, when AMP levels rise. AMPK phosphorylates TSC2 and Raptor, thereby suppressing mTORC1 activity (130, 131). High glycaemic load diets reduce AMPK activity and so stimulate mTORC1 signalling.

Remarkably, in response to AA depletion, mTORC1 activity is rapidly abolished (132). AA starvation impairs binding of mTORC1 to Rheb (133). Of all essential AAs, leucine exerts the greatest effects on mTORC1 signalling (100, 103, 132). AAs promote the translocation of inactive mTORC1 to lysosomal compartments enriched in activated Rheb (122, 123). This spatial regulation of inactive mTORC1 by AAs is mediated by an active Rag heterodimer and is of crucial importance for AA sensing and activation of mTORC1 (134). The pentameric Ragulator complex acts as a scaffold for the Rag GTPases and mTORC1 at the lysosomal membrane. AA accumulation in the lysosomal lumen generates an activating signal that is transmitted in a vacuolar H^+^-ATPase (v-ATPase)-dependent fashion to activate the guanine nucleotide exchange factor (GEF) activity of Ragulator towards RagA. Upon RagA-GTP loading, mTORC1 is recruited to the lysosomal surface where it interacts with Rheb and becomes activated (134). Thus, mTORC1 integrates IIS and energy-derived signals to Rheb, but in parallel requires sufficient AAs for maximal mTORC1 activity (135) (Fig.1). Recent evidence has been provided that proton-assisted AA transporters (PATs) localised on late endosomes and lysosomes (LEL) interact with Rags and are required for mTORC1 activation (136). The v-ATPase interacts with the activated Rag/Ragulator complex to control AA-dependent mTORC1 activation, which is regulated by the rapid accumulation of extracellular AAs in LELs (137). Thus, in response to AAs these molecules form a signalling complex that has been called the ‘nutrisome’ (137). Cycling of protons through this nutrisomal engine induces conformational changes that activate mTORC1, leading to increased translation and cell growth. Importantly, signalling from the insulin receptor and subsequent activation of the PI3K/Akt/Rheb cascade promotes shuttling of PATs from the cell surface to LEL membranes, hence increasing PAT-dependent mTORC1 activation and cell growth (137). In addition, the accumulation of AAs in the LEL lumen presumably involves transport into intracellular endosomal compartments via currently unknown AA transporters (AATs) or potentially via endocytosis. Cytoplasmic leucine, which may be brought into cells via the heterodimeric amino acid transporter CD98 (138), has been shown to play a key role in activating mTORC1 in some cultured cells and may be important in this process. Influx of leucine or other AAs into the LEL system may ultimately allow the AA substrates of PAT1 to accumulate in the LELs through AA exchange mechanisms, leading to PAT1-mediated activation of the nutrisome (136).

WD characterized by high glycaemic load and dairy products results in enhanced IIS and provides abundant energy for mTORC1 activation. High consumption of milk and dairy proteins persistently oversupplied by WD provide abundant amounts of insulinotropic branched-chain AAs (leucine, isloleucine and valine), which enhance mTORC1 signalling. Maximal mTORC1 activity is reached when high IIS, nutrient-derived energy and high AA availability are combined, the typical nutritional constellation provided by WD.

**FoxO1: mTORC1-regulator of keratinocyte and sebocyte proliferation**

Acne is an “anabolic”, hyperproliferative disorder of the pilosebaceous follicle characterized by exaggerated proliferation of acroinfundibular keratinocytes and increased sebocyte growth. Enhanced sebum production indicates increased mTORC1 activation (139, 140). Furthermore, the PI3K/Akt/mTORC1 pathway has been shown to stimulate keratinocyte proliferation (141). Loss-of-function of either the TSC1 or TSC2 gene leads to persistent activation of mTORC1 resulting in the hamartoma syndrome tuberous sclerosis complex (142). The tuberous sclerosis complex exhibits folliculocystic and collagen hamartoma with multiple comedones and keratin-containing cysts lined by infundibular epithelium (143). Acne vulgaris of adolescents of Western societies has recently been proposed to represent an mTORC1-driven disease promoted by exaggerated diet-induced IIS leading to decreased expression of FoxO1-regulated genes and changes of interactions of FoxO1 with other key regulatory proteins of cell signalling (5, 43).

**FoxO1/p21 interaction.** Comedogenesis is associated with increased proliferation of acroinfundibular keratinocytes (139). The comedo is the morphological result of increased keratinocyte proliferation leading to failure of keratinocyte maturation and failed desquamation. Cell proliferation is controlled by two major cell cycle inhibitors: p21 and p27 (146). Withdrawal from the cell cycle and growth arrest are an intrinsic part of the keratinocyte terminal differentiation program (145, 146). It is well established that induction of p21 is the earliest regulatory step associated with epidermal keratinocyte differentiation (147). Notably, p21 is a FoxO1 target gene and FoxO1 induces the expression of p21 (51). Thus, high IIS of WD may reduce FoxO1-mediated p21 expression of keratinocytes and may thus allow uncontrolled proliferation of acroinfundibular keratinocytes already stimulated by growth factors (androgens, IGF-1) during the pubertal growth period.

Isotretinoin, the most potent systemic anti-acne drug, inhibits proliferation of epidermal keratinocytes (148-150). This suggests a therapeutic isotretinoin-FoxO1-p21 interaction. In fact, isotretinoin-induced apoptosis and growth arrest of SEB-1 sebocytes has been associated with increased expression of p21 (151). Thus, accumulating evidence supports the hypothesis that isotretinoin´s pharmacological mode of action is primarily linked to isotretinoin-mediated upregulation of nuclear FoxO1 levels (69).

Sebocytes are highly specialized, sebum producing epithelial cells that release their content by rupture of the cell membrane and cellular degradation (152). Sebocytes are most commonly found in the skin in association with hair follicles, where they arise from stem cell derived cells in the basilar layer of the fetal appendageal bud (152-154). Sebocyte formation and function is controlled by multiple pathways: the transcription factors Sox9, Blimp1, BMP, sonic hedgehog, c-Myc and Wnt/β-catenin play the most important roles in the regulation of SG development (152).

**FoxO1/c-Myc-interaction.** Overexpression of c-Myc in transgenic mice results in enlarged and more numerous SGs at the expense of the hair follicle lineage (155, 156). Furthermore, skin-specific deletion of c-Myc negatively affects SG development (157). Remarkably, recent evidence points to the role of FoxO proteins as regulators of p21-activated kinase-1 (PAK1) expression (158). Both PAK1 and PAK2 are known to inhibit c-Myc by specific phosphorylation (159, 160). Thus, high IIS of WD causing lowered nuclear FoxO levels may result in less PAK-mediated c-Myc inhibition thereby stimulating c-Myc-driven sebocyte proliferation. Thus, the impact of nutritional status, via FoxO1/p21- and FoxO/PAK-mediated regulation of cell cycle control, modifies keratinocyte as well as sebocyte growth and proliferation.

**FoxO1/β-catenin-interaction.** Wnt/β-catenin signalling blocks differentiation towards the sebocyte phenotype, since inhibition of Wnt target genes promotes sebocyte development (161-163). Induction of β-catenin degradation in the skin of developing mice by forced expression of Smad7 perturbed hair follicle morphogenesis and differentiation but accelerated SG morphogenesis (163). In skin, c-Myc and β-catenin appear to exert opposing effects on sebocyte differentiation. Analysis of transgenic mice with simultaneous activation of c-Myc and β-catenin revealed mutual antagonism: c-Myc blocked β-catenin-mediated formation of ectopic hair follicles and β-catenin reduced c-Myc-stimulated sebocyte differentiation (164, 165). Thus, the question arose whether FoxO1 may modify β-catenin signalling and vice versa.

Intriguingly, it has been demonstrated in mammalian cells that β-catenin strongly interacts with FoxO1 and FoxO3a (166). This interaction requires armadillo repeats 1 to 8 of β-catenin and the C-terminal half of FoxO proteins (166). Importantly, binding of β-catenin to FoxO enhances the transcriptional activity of FoxO (166). Thus, increased Wnt/β-catenin-signalling with enhanced nuclear FoxO1 activity may suppress c-Myc-dependent sebocyte growth via activation of the FoxO1-PAK1 pathway. In contrast, ageing has recently been demonstrated to be associated with decreased Wnt signalling (167), which may explain SG hyperplasia observed in elderly individuals. This is compatible with the fact that high nuclear levels of β-catenin bind to FoxO3a and FoxO1 and augment their transcriptional proapoptotic effects (36-38). Furthermore, it has been demonstrated that FoxOs and Tcf factors compete for the limited nuclear pool of β-catenin (168, 169). These observations confirm the pivotal role of the evolutionarily conserved FoxO/β-catenin interactions and provide new insights into the complex signalling network of FoxO1, c-Myc and Wnt/β-catenin signalling in SG development and homeostasis (Table 2).

The canonical Wnt pathway is highly dependent on the activity of glycogen synthase kinase 3 (GSK3). In the absence of Wnt signals β-catenin is phosphorylated by casein kinase 1 and GSK3 triggering β-catenin phosphorylation, ubiquitylation and proteasomal degradation. In the presence of Wnt signals the β-catenin degradation complex including GSK3 is inactivated and β-catenin phosphorylation is blocked allowing its translocation into the nucleus to stimulate the transcription of Wnt target genes (170).

**GSK3/FoxO1/mTORC1-interaction.** Increased IIS, via activation of Akt, triggers not only inhibitory phosphorylation of FoxO1 but also of GSK3 (171, 172). When active under fasting conditions, GSK3 is dephosphorylated (173). Active GSK3 directly phosphorylates and activates TSC2 when primed by AMPK-dependent phosphorylation and inhibits mTORC1 activation (174). Thus, TSC2 integrates Wnt and energy signals via coordinated phosphorylation by AMPK and GSK3 to regulate cell growth (174). In the postprandial state, i.e., conditions of high glucose availability and high insulin signalling, Akt-driven inactivation of GSK3 alleviates TSC2-driven inhibition of Rheb and thus results in mTORC1 activation (174). Recently, direct interaction between FoxO1 and GSK3β has been observed (175). Overexpression of GSK3 augmented FoxO1-mediated glucose-6-phosphatase (G6Pase) promoter activation in a manner dependent on its kinase activity, whereas GSK3 inhibition abolished the G6Pase promoter activation by FoxO1 (175) (Table 2).

Taken together, substantial evidence underlines the central role of the nutrient-sensitive transcription factor FoxO1 as a relaying system orchestrating various important signalling pathways in the pathogenesis of acne. FoxO1 activity is primarily regulated by IIS, interacts at multiple regulatory levels with mTORC1 signalling and modifies pivotal pathways important for sebocyte and keratinocyte proliferation and homeostasis. FoxO1 maintains a close molecular crosstalk with canonical growth-regulating pathways, modifies the expression of FoxO1 target genes, and physically interacts with PPARγ, LXRα, AR, β-catenin and GSK3, most important regulators in SG development and homeostasis (Table 2) (60, 61, 74, 75, 166, 175).

**TNFα-stimulates mTORC1 signalling**

T cells constantly monitor energy status and nutrient levels in order to adjust metabolic pathways according to their nutritional status (176). The regulation of cellular metabolism is tightly coupled to T cell differentiation. mTORC1 has emerged as a key player in sensing nutritional/energetic signals. The mTORC1 pathway plays an important role in determining the differentiation of CD4(+) T cells into inflammatory and regulatory subsets, in the induction of anergy, in the development of CD8(+) memory T cells and the regulation of T cell trafficking (176). Inflammation is a highly energy-consuming process and T-cell trafficking as well as T-cell expansion requires the activation of mTORC1 (177). Antigen recognition by naive CD4(+) and CD8(+) T cells triggers mTORC1 activation, which in turn programmes the differentiation of these cells into functionally distinct lineages (178). Thus, mTORC1 provides a vital link between immune function and metabolism (175). The findings explain why rapamycin, a potent inhibitor of mTORC1, exerts immunosuppressive activities.

There is accumulating evidence that genetic variants of the TNFα-gene (*TNF*) increase the susceptibility for inflammatory acne (179-181). Enhanced TNFα signalling increases the activity of the kinase IKKβ, which by suppression of TSC1 activates mTORC1 signalling (182, 183). Proinflammatory signals of TNFα thus increase mTORC1 activity, which via lipin-1 phosphorylation controls nuclear activity of SREBP-1 and thereby induces lipogenesis (110, 184). In fact, it has recently been demonstrated that TNF-α stimulated lipogenesis in SZ95 human sebocytes (95). However, TNF-α could not induce both expression of lipogenic proteins and lipid synthesis when Akt expression was attenuated (95). Akt plays a crucial role for TSC2 phosphorylation, which activates Rheb-mediated activation of mTORC1 (96). TNFα-stimulated mTORC1-driven SG lipogenesis and proliferation of acroinfundibular keratinocytes may thus represent the molecular mechanism of some subsets of hereditary acne due to *TNF* polymorphisms with increased TNFα/IKKβ/mTORC1 signalling (Fig. 1S).

Over-activated TNFα/IKKβ/TSC1/Rheb/mTORC1 signalling in individuals with acne-promoting *TNF* polymorphisms may explain the occurrence of high-grade inflammatory acne with intensive matrix metalloproteinase-mediated tissue destruction. Thus, conglobate and fulminant acne may benefit from treatment with TNFα-antagonists.

The green tea polyphenol epigallocatechin-3-gallate (EGCG) is a natural mTORC1 kinase inhibitor (185) and should thus be able to attenuate mTORC1/lipin-1/SREBP-1-driven lipogenesis. Indeed, a 3% green tea emulsion reduced skin sebum production in male volunteers (186). Intriguingly, topical application of EGCG to rabbit auricles reduced the SG size (187). Notably, mTORC1 is the most important regulator that determines cell size and proliferation (110). When applied to cultured human SZ95 sebocytes, EGCG strongly suppressed cell proliferation and lipogenesis and decreased the expression of proinflammatory cytokines IL-1, IL-6, and IL-8 (187). These data underline the pivotal role of well-balanced mTORC1 signalling to maintain SG homeostasis.

**Vitamin D activates FoxO signalling**

Recently, sebocytes were identified as 1,25-dihydroxyvitamin D3 (1,25D3)- responsive target cells, indicating that vitamin D analogues may be effective in the treatment of acne (188, 189). In keratinocytes and other cell types, 1,25D3 signals through the nuclear vitamin D receptor (VDR) and regulates growth and differentiation (188). Accumulating data have revealed that 1,25D3 and FoxO proteins similarly regulate common target genes. In fact, ligand-bound VDR regulates the posttranslational modification and function of FoxO proteins (190). 1,25D3 treatment enhances binding of FoxO3a and FoxO4 to promoters of FoxO target genes and blocks growth factor-induced FoxO protein nuclear export. Furthermore, VDR associates directly with FoxO proteins and regulators, the sirtuin 1 (Sirt1) class III histone deacetylase (HDAC), and protein phosphatase 1. 1,25D3 treatment rapidly induced FoxO deacetylation and dephosphorylation, consistent with FoxO activation (190). In contrast, ablation of VDR expression enhanced FoxO3a phosphorylation. 1,25D3-dependent cell cycle arrest was blocked in FoxO3a-deficient cells, indicating that FoxO proteins are key downstream mediators of the antiproliferative actions of 1,25D3. These observations link 1,25D3 signalling through VDR directly to FoxO function and provide the molecular basis for the synergistic antiproliferative and anti-inflammatory signalling of vitamin D and FoxOs (Fig. 1S).

Remarkably, acne often clinically improves during summer time when systemic vitamin D levels are elevated. Comedolytic effects of topically applied active vitamin D3 analogues have been demonstrated on pseudocomedones in the rhino mouse (191, 192). In addition, oral isotretinoin treatment of acne patients significantly increased serum levels of 1,25D3 (193). Increased 1,25D3 may thus further augment VDR-mediated nuclear expression of FoxO proteins. Furthermore, isotretinoin-mediated increase of 1,25D3 serum levels may explain the risk for skeletal hyperostoses after prolonged high-dose isotretinoin treatment.

**Vitamin D inhibits mTORC1 signalling**

Vitamin D not only activates FoxO signalling, but is a strong inhibitor of mTORC1. It has recently been recognized that 1,25D3 stimulates the mRNA and protein expression of *DNA damage-inducible transcript 4* (DDIT4; also known as DNA damage response 1, REDD1) (194). DDIT4 knockdown by siRNA completely suppressed the antiproliferative effects of 1,25D3 (195). DDIT4 facilitates the assembly and activation of TSC1/TSC2 complex for eventual suppression of downstream mTORC1 activity (194). mTORC1 thus appears to be a master regulator target for the immunomodulatory and antiproliferative effects of vitamin D (Fig. 1S).

Vitamin D exerts further inhibitory effects on mTORC1 signalling. Vitamin D at the promoter level induces MAPK phosphatase-1 (MKP-1) (196-198), which is a most important feedback inhibitor of monocytes/macrophages during activated states of innate immune responses (199-201). Vitamin D/MKP-1-mediated attenuation of innate immunity may thus ameliorate TNFα signalling, which may lower mTORC1 activity via decreased TNFα/IKKβ/TSC1 signalling.

**Acne-promoting gene polymorphisms activate mTORC1**

Several genetic aberrations like *TNF*- and *IGF1* polymorphisms and *AR polymorphisms with shorter CAG repeat lengths* have been associated with increased acne risk. There is convincing evidence that these genetic polymorphisms share a common functional signalling abnormality: the enhanced stimulation of mTORC1.

***TNF* gene polymorphisms** associated with increased risk of acne (179-181) may induce acne-promoting effects by increased IKKβ-mediated phosphorylation of TSC1 (182, 183). This may result in increased Rheb-mediated activation of mTORC1 (Fig. 1S)

**Inherited *AR CAG* polymorphisms with shorter CAG repeats** lead to enhanced androgen signalling. In these individuals a higher predisposition for acne has been reported (202-204). AR signalling activates mTORC2 (77), which activates Akt and thereby enhances mTORC1 signalling. Moreover, AR/mTORC2-mediated activation of Akt decreases the nuclear content of the AR cosuppressor FoxO1 by Akt-mediated nuclear export of FoxO1 (73-77)(Fig. 1S).

***IGF1* polymorphism** has recently been associated with increased acne risk (205). Enhanced IGF-1 signalling via hyperactivation of Akt stimulates mTORC1 activity and may thus explain the increased risk for acne. These individuals may be more susceptible to IGF-1 enhancing effects of WD. In fact, Jung *et al.* (23) demonstrated that acne patients who observed aggravation of their acne by food consumption exhibited significant higher IGF-1 serum levels (543.9 ng/mL) than those with lower serum IGF-1 levels (391.3 ng/mL) who did not observe food-aggravation of their acne. In contrast, individuals with congenital IGF-1 deficiency due to a GHR defect never develop acne and other mTORC1-driven diseases of civilization and exhibit higher nuclear FoxO activity and lower mTORC1 activity (Fig. 1S)(47, 28).

These observations clearly underline the pivotal impact of FoxO1-/mTORC1-controlled nutrient signalling especially in the context of hereditable genetic abnormalities, which contribute to acne pathogenesis. They all appear to converge downstream in FoxO1/mTORC1-mediated signalling processes which may increase follicular keratinocyte proliferation, stimulate SG hyperplasia with enhanced sebaceous lipogenesis, promote chronic follicular and perifollicular inflammation with activated innate and adaptive immunity with deviant AMP expression, which may allow *P. acnes* overgrowth and pathogenic biofilm formation.

**References**

**122** Sancak Y, Peterson T R, Shaul Y D *et al.* The Rag GTPases bind raptor and mediate amino acid signaling to mTORC1. Science 2008: **320:** 1496-1501.

**123** Sancak Y, Bar-Peled L, Zoncu R *et al*. Ragulator-Rag complex targets mTORC1 to the lysosomal surface and is necessary for its activation by amino acids. Cell 2010: **141:** 290-303.

**124** Goberdhan D C. Intracellular amino acid sensing and mTORC1-regulated growth: New ways to block an old target? Curr Opin Invest Drugs 2010: **11:** 1360-1367.

**125** Lee D F, Kuo H P, Chen C T *et al.* [IKK beta suppression of TSC1 links inflammation and tumor angiogenesis via the mTOR pathway.](http://www.ncbi.nlm.nih.gov/pubmed/17693255) Cell 2007: **130:** 440-455.

**126** Dan H C, Cooper M J, Cogswell P C *et al*. [Akt-dependent regulation of NF- {kappa}B is controlled by mTOR and Raptor in association with IKK.](http://www.ncbi.nlm.nih.gov/pubmed/18519641) Genes Dev 2008: **22:** 1490-1500.

**127** Inoki K, Li Y, Zhu T *et al.* TSC2 is phosphorylated and inhibited by Akt and suppresses mTOR signalling. Nat Cell Biol 2002: **4:** 648-657.

**128** Manning B D, Tee A R, Logsdon M N *et al.* Identification of the tuberous sclerosis complex-2 tumor suppressor gene product tuberin as a target of the phosphoinositide-3-kinase/akt pathway. Mol Cell 2002: **10:** 151-162.

**129** Tee A R, Fingar D C, Manning B D *et al.* Tuberous sclerosis complex-1 and -2 gene products function together to inhibit mammalian target of rapamycin (mTOR)-mediated downstream signaling. Proc Natl Acad Sci USA 2002: **99:** 13571-13576.

**130** Inoki K, Zhu T, Guan K L. TSC2 mediates cellular energy response to control cell growth and survival. Cell 2003: **115:** 577-590.

**131** Gwinn D M, Shackelford D B, Egan D F *et al.* AMPK phosphorylation of raptor mediates a metabolic checkpoint. Mol Cell 2008: **30:** 214-226.

**132** Hara K, Yonezawa K, Weng Q P *et al.* Amino acid sufficiency and mTOR regulate p70 S6 kinase and eIF-4EBP1 through a common effector mechanism. J Biol Chem 1998: **273:** 14484-14494.

**133** Long X, Ortiz-Vega S, Lin Y *et al.* Rheb binding to mammalian target of rapamycin (mTOR) is regulated by amino acid sufficiency. J Biol Chem 2005: **280:** 23433-23436.

**134** Bar-Peled L, Schweitzer L D, Zoncu R *et al*. Ragulator is a GEF for the Rag GTPases that signal amino acid levels to mTORC1. Cell 2012: **150:** 1196-1208.

**135** [Nobukuni T](http://www.ncbi.nlm.nih.gov/pubmed?term="Nobukuni T"%5BAuthor%5D), Joaquin M, Roccio M *et al.* Amino acids mediate mTOR/raptor signaling through activation of class 3 phosphatidylinositol 3OH-kinase. Proc Natl Acad Sci USA 2005: **102:** 14238-14243.

**136** Ögmundsdóttir M H, Heublein S, Kazi S *et al.* Proton-assisted amino acid transporter PAT1 complexes with Rag GTPases and activates TORC1 on late endosomal and lysosomal membranes. PLoS ONE 2012: **7:** e36616.

**137** Zoncu R, Bar-Peled L, Efeyan A *et al.* mTORC1 senses lysosomal amino acids through an inside-out mechanism that requires the vacuolar H- ATPase. Science 2011: **334:** 678-683.

**138** Reynolds B, Laynes R, Ögmundsdóttir M H *et al*. Amino acid transporters and nutrient-sensing mechanisms: new targets for treating insulin-linked disorders? Biochem Soc Transact 2007: **35:** 1215-1217.

**139** Plewig G, Fulton J E, Kligman A M. Cellular dynamics of comedo formation in acne vulgaris. Arch Dermatol Forsch 1971: **242:** 12-29.

**140** [Zouboulis C C](http://www.ncbi.nlm.nih.gov/pubmed?term=Zouboulis CC%5BAuthor%5D&cauthor=true&cauthor_uid=9557220), [Xia L](http://www.ncbi.nlm.nih.gov/pubmed?term=Xia L%5BAuthor%5D&cauthor=true&cauthor_uid=9557220), [Akamatsu H](http://www.ncbi.nlm.nih.gov/pubmed?term=Akamatsu H%5BAuthor%5D&cauthor=true&cauthor_uid=9557220) *et al.* The human sebocyte culture model provides new insights into development and management of seborrhoea and acne. Dermatology 1998: **196:** 21-31.

**141** Squarize C H, Castilho R M, Bugge T H *et al.* [Accelerated wound healing by mTOR activation in genetically defined mouse models.](http://www.ncbi.nlm.nih.gov/pubmed/20498714) PLoS One 2010: **5:** e10643.

**142** Rosner M, Hanneder M, Siegel N *et al.* The mTOR pathway and its role in human genetic diseases. Mutation Res 2008: **659:** 284-292.

**143** [Torrelo A](http://www.ncbi.nlm.nih.gov/pubmed?term=Torrelo A%5BAuthor%5D&cauthor=true&cauthor_uid=21839539), [Hadj-Rabia S](http://www.ncbi.nlm.nih.gov/pubmed?term=Hadj-Rabia S%5BAuthor%5D&cauthor=true&cauthor_uid=21839539), [Colmenero I](http://www.ncbi.nlm.nih.gov/pubmed?term=Colmenero I%5BAuthor%5D&cauthor=true&cauthor_uid=21839539) *et al.* Folliculocystic and collagen hamartoma of tuberosus sclerosis complex. J Am Acad Dermatol 2011: **66:** 617-621.

**144** [Yoon M K](http://www.ncbi.nlm.nih.gov/pubmed?term=Yoon MK%5BAuthor%5D&cauthor=true&cauthor_uid=22988851), [Mitrea D M](http://www.ncbi.nlm.nih.gov/pubmed?term=Mitrea DM%5BAuthor%5D&cauthor=true&cauthor_uid=22988851), [Ou L](http://www.ncbi.nlm.nih.gov/pubmed?term=Ou L%5BAuthor%5D&cauthor=true&cauthor_uid=22988851) *et al.* Cell cycle regulation by the intrinsically disordered proteins p21 and p27. Biochem Soc Trans 2012: **40:** 981-988.

**145** Missero C, Calautti E, Eckner R *et al.* Involvement of the cell cycle inhibitor Cip1/WAF1 and the E1A-associated p300 protein in terminal differentiation. Proc Natl Acad Sci USA 1995: **92:** 5451-5455.

**146** Missero C, Di Cunto F, Kiyokawa H *et al.* The absence of p21*^Cip1/WAF1^* alters keratinocyte growth and differentiation and promotes ras-tumor progression. Genes Dev 1996: **10:** 3065-3075.

**147** Rangarajan A, Talora C, Okuyama R *et al.* Notch signaling is a direct determinant of keratinocyte growth arrest and entry into differentiation. EMBO J 2001: **20:** 3427-3436.

**148** Marcelo C L, Madison K C. Regulation of the expression of epidermal keratinocyte proliferation and differentiation by vitamin A analogs*.* Arch Dermatol Res 1984: **276:** 381-389.

**149** Schroeder M, Zouboulis C C. All-trans-retinoic acid and 13-cis-retinoic acid: pharmacokinetics and biological activity in different cell culture models of human keratinocytes. Horm Metab Res 2007: **39:** 136-140.

**150** Popadic S, Ramic Z, Medenica L *et al*. Antiproliferative effect of vitamin A and D analogues on adult human keratinocytes in vitro. Skin Pharmacol Physiol 2008: **21:** 227-234.

**151** Nelson A M, Gilliland K L, Cong Z *et al.* 13-cis retinoic acid induces apoptosis and cell cycle arrest in human SEB-1 sebocytes. J Invest Dermatol 2006: **126:** 2178-2189.

**152** Schneider M R, Paus R. Sebocytes, multifaceted epithelial cells: Lipid production and holocrine secretion. Int J Biochem Cell Biol 2009: **42:** 181-185.

**153** [Zouboulis C C](http://www.ncbi.nlm.nih.gov/pubmed?term=Zouboulis CC%5BAuthor%5D&cauthor=true&cauthor_uid=15556719). Acne and sebaceous gland function. Clin Dermatol 2004: **22:** 360-366.

**154** [Zouboulis C C](http://www.ncbi.nlm.nih.gov/pubmed?term=Zouboulis CC%5BAuthor%5D&cauthor=true&cauthor_uid=18474083), [Baron J M](http://www.ncbi.nlm.nih.gov/pubmed?term=Baron JM%5BAuthor%5D&cauthor=true&cauthor_uid=18474083), [Böhm M](http://www.ncbi.nlm.nih.gov/pubmed?term=Böhm M%5BAuthor%5D&cauthor=true&cauthor_uid=18474083) *et al.* Frontiers in sebaceous gland biology and pathology. Exp Dermatol 2008: **17:** 542-551.

**155** Arnold I, Watt F M. c-Myc activation in transgenic mouse epidermis results in mobilization of stem cells and differentiation of their progeny. Curr Biol 2001: **11:** 558-568.

**156** Waikel R L, Kawachi Y, Waikel P A *et al.* Deregulated expression of c-Myc depletes epidermal stem cells. Nat Genet 2001: **28:** 165-168.

**157** Zanet J, Pibre S, Jacquet C *et al*. Endogenous Myc controls mammalian epidermal cell size, hyperproliferation, endoreplication and stem cell amplification. J Cell Sci 2005: **118:** 1693-1704.

**158** de la Torre-Ubieta L, Gaudilliere B, Yang Y *et al*. A FOXO-Pak1 transcriptional pathway controls neuronal polarity. Genes Dev 2010: **24:** 799-813.

**159** Ong C C, Jubb A M, Zhou W *et al.* p21-activated kinase 1: PAK´ed with potential. Oncotarget 2011: **2:** 491-492.

**160** Berta M A, Baker C M, Cittle D L *et al.* Dose and context dependent effects of Myc on epidermal stem cell proliferation and differentiation. EMBO Mol Med 2009: **2:** 16-25.

**161** Merrill B J, Gat U, DasGupta R *et al*. Tcf3 and Lef1 regulate lineage differentiation of multipotent stem cells in skin. Genes Dev 2001: **15:** 1688-1705.

**162** Niemann C, Owens D M, Hulsken J *et al.* Expression of DeltaNLef1 in mouse epidermis results in differentiation of hair follicles into squamous epidermal cysts and formation of skin tumours. Development 2002: **129:** 95- 109.

**163** Han G, Li A G, Liang Y Y *et al.* Smad7-induced beta-catenin degradation alters epidermal appendage development. Dev Cell 2006: **11:** 301-312.

**164** Niemann C. Differentiation of the sebaceous gland. Dermatoendocrinol 2009: **1:** 64-67.

**165** Lo Celso C, Berta M A, Braun K M *et al.* Characterization of bipotent epidermal progenitors derived from human sebaceous gland: Contrasting roles of c-myc and β-catenin. Stem Cells 2008: **26:** 1241-1252.

**166** Essers M A, de Vries-Smits L M, Barker N *et al.* Functional interaction of beta- catenin and FOXO in oxidative stress signaling. Science 2005: **308:** 1181-1184.

**167** Makrantonaki E, Brink T C, Zampeli V *et al.* Identification of biomarkers of human skin ageing in both genders. Wnt signalling – a label of skin ageing? PLoS ONE 2012: **7:** e50393.

**168** Jin T, Fantus G I, Sun J. Wnt and beyond Wnt: multiple mechanisms control the transcriptional property of β-catenin. Cell Signal 2008: **20:** 1697- 1704.

**169** Hoogeboom D, Essers M A , Polderman P E *et al.* Interaction of FOXO with β- catenin inhibits β-catenin/T cell factor activity. J Biol Chem 2008: **283:** 9224- 9230.

**170** Wu D, Pan W. GSK3: a multifaceted kinase in Wnt signaling. Trends Biochem Sci 2010: **35:** 161-168.

**171** Liang J, Slingerland J M. Multiple roles of the PI3K/PKB (Akt) pathway in cell cycle progression. Cell Cycle 2003: **2:** 339-345.

**172** Cross D A, Alessi D R, Cohne P *et al.* Inhibition of glycogen synthase kinase-3 by insulin mediated by protein kinase B. Nature 1995: **378:** 785- 789.

**173** Patel S, Doble B W, MacAulay K *et al.* Tissue-specific role of glycogen synthase kinase 3β in glucose homeostasis and insulin action. Mol Cell Biol 2008: **28:** 6314-6328.

**174** [Inoki K](http://www.ncbi.nlm.nih.gov/pubmed?term=Inoki K%5BAuthor%5D&cauthor=true&cauthor_uid=16959574), [Ouyang H](http://www.ncbi.nlm.nih.gov/pubmed?term=Ouyang H%5BAuthor%5D&cauthor=true&cauthor_uid=16959574), [Zhu T](http://www.ncbi.nlm.nih.gov/pubmed?term=Zhu T%5BAuthor%5D&cauthor=true&cauthor_uid=16959574) *et al*. TSC2 integrates Wnt and energy signals via a coordinated phosphorylation by AMPK and GSK3 to regulate cell growth. Cell 2006: **126:** 955-968.

**175** Sakamaki J I, Daitoku H, Kaneko Y *et al.* GSK3β regulates gluconeogenic gene expression through HNF4α and FOXO1. J Recept Signal Transduct Res 2012: **32:** 96-101.

**176** Peter C, Waldmann H Cobbold S P. mTOR signalling and metabolic regulation of T cell differentiation. Curr Opin Immunol 2010: **22:** 655-661.

**177** Powell J D, Pollizzi K N, Heikamp E B *et al.* Regulation of immune responses by mTOR. Annu Rev Immunol 2012: **30:** 39-68.

**178** Chi H. Regulation and function of mTOR signalling in T cell fate decisions. Nat Rev Immunol 2012: **12:** 325-338.

**179** Szabó K, Tax G, Teodorescu-Brinzeu D *et al.* TNFα gene polymorphisms in the pathogenesis of acne vulgaris. Arch Dermatol Res 2011: **303:** 19-27.

**180** Szabó K, Kemény L. Studying the genetic predisposing factors in the pathogenesis of acne vulgaris. Hum Immunol 2011: **72:** 766-773.

**181** Al-Shobaili H A, Salem T A, Alzolibani A A *et al.* Tumor necrosis factor-α -308 G/A and interleukin 10 -1082 A/G gene polymorphisms in patients with acne vulgaris. J Dermatol Sci 2012: **68**: 52-55.

**182** Lee D F, Kuo H P, Chen C T *et al.* [IKK beta suppression of TSC1 links inflammation and tumor angiogenesis via the mTOR pathway.](http://www.ncbi.nlm.nih.gov/pubmed/17693255) Cell 2007: **130:** 440-455.

**183** Dan H C, Cooper M J, Cogswell P C *et al.* [Akt-dependent regulation of NF- {kappa}B is controlled by mTOR and Raptor in association with IKK.](http://www.ncbi.nlm.nih.gov/pubmed/18519641) Genes Dev 2008: **22:** 1490-1500.

**184** Peterson T R, Sengupta S S, Harris T E *et al.* mTOR complex 1 regulates lipin 1 localization to control the SREBP pathway. Cell 2011: **146:** 408-420.

**185** Van Aller G S, Carson JD, Tang W *et al.* Epigallocatechin gallate (EGCG), a major component of green tea, is a dual phosphoinositide-3-kinase/mTOR inhibitor. Biochem Biophys Res Commun 2011: **406:** 194-199.

**186** [Mahmood T](http://www.ncbi.nlm.nih.gov/pubmed?term="Mahmood T"%5BAuthor%5D), [Akhtar N](http://www.ncbi.nlm.nih.gov/pubmed?term="Akhtar N"%5BAuthor%5D), [Khan B A](http://www.ncbi.nlm.nih.gov/pubmed?term="Khan BA"%5BAuthor%5D) *et al*. Outcomes of 3% green tea emulsion on skin sebum production in male volunteers. Bosn J Basic Med Sci 2010: **10:** 260-264.

**187** [Im M](http://www.ncbi.nlm.nih.gov/pubmed?term=Im M%5BAuthor%5D&cauthor=true&cauthor_uid=22763784), [Kim S Y](http://www.ncbi.nlm.nih.gov/pubmed?term=Kim SY%5BAuthor%5D&cauthor=true&cauthor_uid=22763784), [Sohn K C](http://www.ncbi.nlm.nih.gov/pubmed?term=Sohn KC%5BAuthor%5D&cauthor=true&cauthor_uid=22763784) *et al.* Epigallocatechin-3-gallate suppresses IGF-I- induced lipogenesis and cytokine expression in SZ95 sebocytes. J Invest Dermatol 2012: **132:** 2700-2708.

**188** Reichrath J. Vitamin D and the skin: an acient friend, revisited. Exp Dermatol 2007: **16:** 618-625.

**189** Reichrath J, Lehmann B, Carlberg C *et al.* Vitamines as hormones. Horm Metab Res 2007: **39:** 71-84.

**190** [An B S](http://www.ncbi.nlm.nih.gov/pubmed?term=An BS%5BAuthor%5D&cauthor=true&cauthor_uid=20733005), [Tavera-Mendoza L E](http://www.ncbi.nlm.nih.gov/pubmed?term=Tavera-Mendoza LE%5BAuthor%5D&cauthor=true&cauthor_uid=20733005), [Dimitrov V](http://www.ncbi.nlm.nih.gov/pubmed?term=Dimitrov V%5BAuthor%5D&cauthor=true&cauthor_uid=20733005) *et al.* Stimulation of Sirt1-regulated FoxO protein function by the ligand-bound vitamin D receptor. Mol Cell Biol 2010: **30:** 4890-4900.

**191** Hayashi N, Watanabe H, Yasukawa H *et al.* Comedolytic effect of topically applied vitamin D3 analogue on pseudocomedones in the rhino mouse. Br J Dermatol 2006: **155:** 895-901.

**192** Nieves N J, Ahrens J M, Plum L A *et al.* Identification of a unique subset of 2- methylene-19-nor analogs of vitamin D with comedolytic activity in the rhino mouse. J Invest Dermatol 2010: **130:** 2359-2367.

**193** Ertugrul D T, Karadag A S, Tutal E *et al.* Does isotretinoin have effect on vitamin D physiology and bone metabolism in acne patients? Dermatol Ther 2011: **24:** 291-295.

**194** Lisse T S, Hewison M. Vitamin D. A new player in the world of mTOR signaling. Cell Cycle 2011: **10:12:** 1888-1889.

**195** Lisse T S, Liu T, Irmler M *et al*. Gene targeting by the vitamin D response element binding protein reveals a role for vitamin D in osteoblast mTOR signaling. FASEB J 2011: **25:** 937-947.

**196** Sutherland E R, Goleva E, Leisa P *et al.* Vitamin D levels, lung function, and steroid response in adult asthma. Am J Respir Crit Care Med 2010: **181**: 699- 704.

**197** Zhang Y, Leung D Y, Richers B N *et al.* Vitamin D inhibits monocyte/ macrophage proinflammatory cytokine production by targeting MAPK phosphatase-1. J Immunol 2012: **188:** 2127-2135.

**198** Griffin A C 3^rd^, Kern M J, Kirkwood K L. MKP-1 is essential for canonical vitamin D-induced signaling through nuclear import and regulates RANKL expression and function. Mol Endocrinol 2012: **26:** 1682-1693.

**199** Wang X, Liu Y. Regulation of innate immune response by MAP kinase phosphatase-1. Cell Signal 2007: **19:** 1372-1382.

**200** Wancket L M, Frazier W J, Liu Y. Mitogen-activated protein kinase phosphatase (MKP)-1 in immunology, physiology, and disease. Life Sci 2012: **90:** 237-248.

**201** Huang G, Chi L Z, Chi H. Regulation of JNK and p38 MAPK in the immune system: signal integration, propagation and termination. Cytokine 2009: **48:** 161-169.

**202** Sawaya M E, Shalita A R. Androgen receptor polymorphism (CAG repeat lengths) in androgenetic alopecia, hirsutism, and acne. J Cutan Med Surg 1998: **3:** 9-15.

**203** Pang Y, He C D, Liu Y *et al.* Combination of short CAG and GGN repeats in the androgen receptor gene is associated with acne risk in North East China. J Eur Acad Dermatol Venereol 2008: **22:** 1445-1451.

**204** Yang Z, Yu H, Cheng B *et al*. Relationship between the CAG repeat polymorphism in the androgen receptor gene and acne in the Han ethnic group. Dermatology 2009: **218:** 302-306.

**205** Tasil L, Turgut S, Kacar N *et al.* Insulin-like growth factor-I gene polymorphism in acne vulgaris. J Eur Acad Dermatol Venereol 2011: doi:10.1111/j.1468-3083.2011.04299.x.

**Figure 1S**

Interaction of acne risk-enhancing gene polymorphisms on FoxO1-/mTORC1 mediated nutrient signalling of Western diet. *TNF* polymorphisms with increased TNFα activity (TNFα*) promote IKKβ-mediated inhibitory TSC1 phosphorylation. *IGF1* polymorphisms with higher IGF-1 activity (IGF-1*) increase Akt-mediated inhibitory phosphorylation of FoxO1 and TSC2. Hyperandrogenaemia, androgen precursors provided by commercial milk consumption or *AR* polymorphism with shorter CAG repeats (AR*) all result in enhanced AR signalling may inhibit TSC2 by increased mTORC2-mediated phosphorylation of Akt. All three genetic abnormalities may over-activate mTORC1 and may thus promote acne. In contrast, 1,25-dihydroxyvitamin D3 (VitD) by increasing the expression of DNA damage-inducible transcript 4 (DDIT4) may inhibit mTORC1 signalling and increase nuclear FoxO activity and may thus exert anti-acne effects. TNFα=tumour necrosis factor-α; IGF1-=insulin-like growth factor-1; LAT= L-type amino acid transporter; TNFR=TNFα receptor; IGF1R=IGF-1 receptor; IR=insulin receptor; AR=androgen receptor; IRS=insulin receptor substrate; PI3K=phosphoinositol-3 kinase; Akt=Akt kinase (protein kinase B); IKKβ=inhibitor of kappa light chain gene enhancer in B cells; FoxO=forkhead box transcription factor class O; TSC=tuberous sclerosis complex; Rheb=ras homolog enriched in brain; mTORC1=mammalian target of rapamycin complex 1; S6K1=S6 kinase 1;

**Abbreviations**

AA=amino acids

Akt=Akt kinase (protein kinase B)

AMP=antimicrobial peptide

AMPK=adenosine monophosphate-activated protein kinase

AR=androgen receptor

ATP=adenosine triphosphate

ATRA=*all-trans*-retinoic acid

Bnip3=Bcl-2/adenovirus E1B 19-kDa-interacting protein 3

COX=cyclooxygenase

CRM1=chromosomal region maintenance protein 1 (=exportin-1)

1,25D3=1,25dihydroxyvitamin D3

DDIT4=DNA damage-inducible transcript 4

EGCG= epigallocatechin-3-gallate

ERK=mitogen activated protein kinase

FoxO= forkhead box class O transcription factor

4E-BP= eukaryotic initiation factor (eIF) 4E-binding protein

GH=growth hormone

GHR=growth hormone receptor

GIP=glucose-dependent insulinotropic polypeptide

GDP=guanosine diphosphate

GSK=glycogen synthase kinase

GTP=guanosine triphosphate

HO-1=heme oxygenase-1

IGF=insulin-like growth factor

IGFBP=IGF binding protein

IGF1R=IGF-1 receptor

IIS=insulin/IGF-1 signalling

IKKβ=inhibitor of kappa light chain gene enhancer in B cells

IL=interleukin

IR=insulin resistance

IRS=insulin recptor substrate

JNK=Jun-N-terminus kinase

LEL=late endosome and lysosome

LKB=liver kinase B

LXR=liver X receptor

MMP=matrix metalloproteinase

mTOR=mammalian target of rapamycin

NF-κB=nuclear factor kappa B

PAK=p21-activated kinase

PI3K=phosphoinositol-3 kinase

PPAR=peroxisome proliferator-activated receptor

PTEN=phosphatase and tensin homolog deleted on chromosome 10

Rag=Ras-related GTP-binding protein

Raptor=regulatory associated protein of mTOR

RAR=retinoic acid receptor

Rheb=Ras homolog enriched in brain

Rictor=rapamycin-insensitive companion of mTOR

RXR=retinoid X receptor

ROS=reactive oxygen species

SG=sebaceous gland

S6K=p70 S6 kinase

SREBP=sterol regulatory element binding protein

RSK=ribosomal S6 kinase

TOR=target of rapamycin

Trb3=tribbles3

TSC=tuberous sclerosis complex

TSC1=hamartin

TSC2=tuberin

WD=Western diet
