# Supplementary material for: Potential role of FoxO1 and mTORC1 in the pathogenesis of Western diet-induced acne
Source: Exp Dermatol. 2013 Apr 25;22(5):311–5. doi: 10.1111/exd.12142 (PMC3746128; doi:10.1111/exd.12142)
Supplement: Supplementary file 2 [file exd0022-0311-SD2.ppt]

## Slide 1
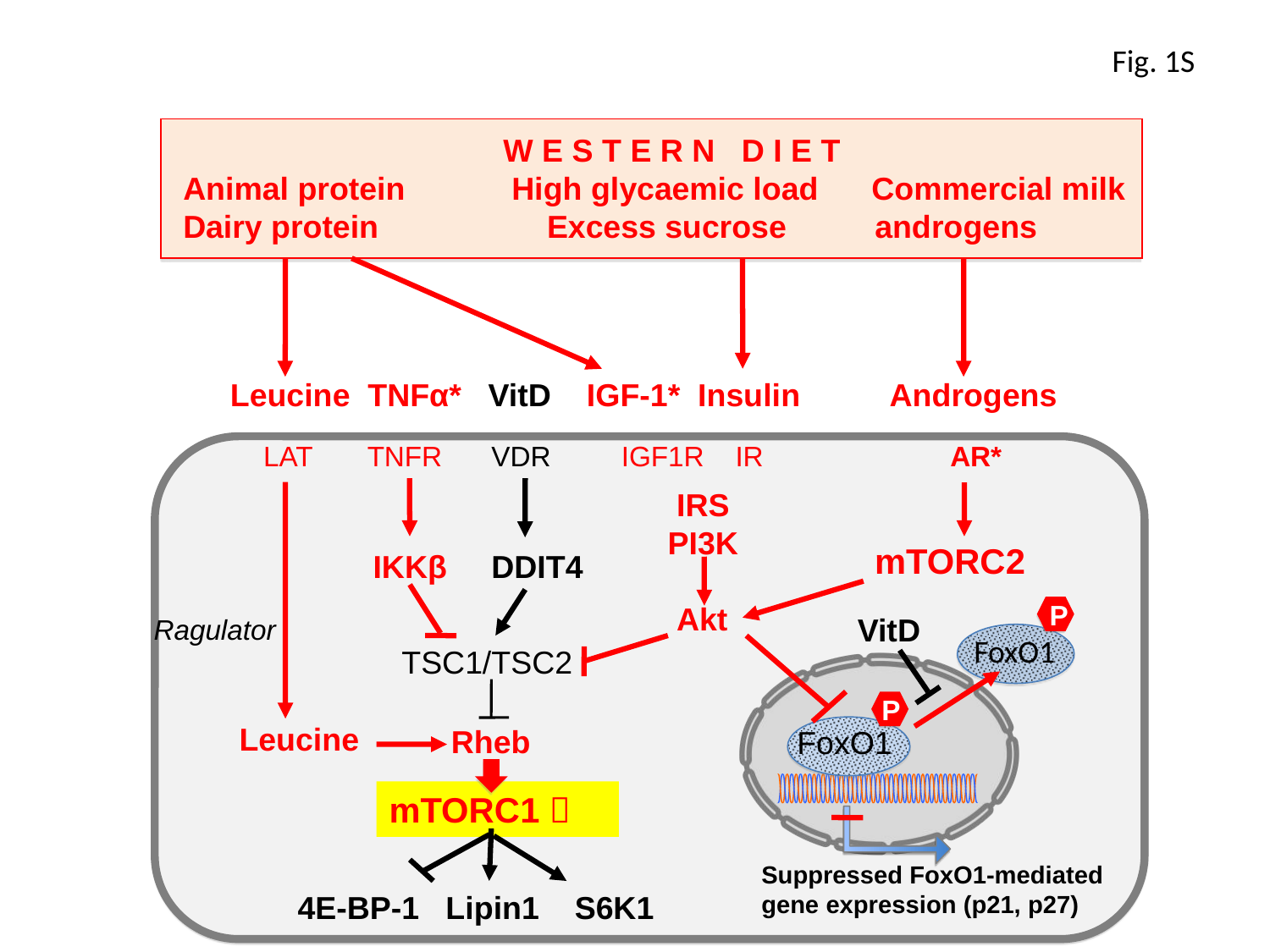

Fig. 1S
 W E S T E R N D I E T
Animal protein High glycaemic load Commercial milk
Dairy protein Excess sucrose androgens
 Leucine TNFα* VitD IGF-1* Insulin Androgens
 LAT TNFR
 VDR IGF1R IR AR*
 IRS
 PI3K
 Akt
 mTORC2
 IKKβ DDIT4
P
 VitD
Ragulator
FoxO1
TSC1/TSC2
P
 Leucine
Rheb
FoxO1
mTORC1 
Suppressed FoxO1-mediated
gene expression (p21, p27)
4E-BP-1 Lipin1 S6K1
